# Using Mobile Health Tools to Assess Physical Activity Guideline Adherence and Smoking Urges: Secondary Analysis of mActive-Smoke

**DOI:** 10.2196/14963

**Published:** 2020-01-06

**Authors:** Rongzi Shan, Lisa R Yanek, Luke G Silverman-Lloyd, Sina Kianoush, Michael J Blaha, Charles A German, Garth N Graham, Seth S Martin

**Affiliations:** 1 Ciccarone Center for the Prevention of Cardiovascular Disease Division of Cardiology, Department of Medicine Johns Hopkins University School of Medicine Baltimore, MD United States; 2 David Geffen School of Medicine at University of California Los Angeles Los Angeles, CA United States; 3 Department of Medicine Johns Hopkins University School of Medicine Baltimore, MD United States; 4 University of California, Berkeley-University of California, San Francisco Joint Medical Program Berkeley, CA United States; 5 Yale New Haven Medical Center - Waterbury Hospital Waterbury, CT United States; 6 Heart and Vascular Center of Excellence Wake Forest Baptist Health Winston-Salem, NC United States; 7 Aetna Foundation Hartford, CT United States

**Keywords:** physical activity, smoking, mHealth, fitness trackers, short message service

## Abstract

**Background:**

Rates of cigarette smoking are decreasing because of public health initiatives, pharmacological aids, and clinician focus on smoking cessation. However, a sedentary lifestyle increases cardiovascular risk, and therefore, inactive smokers have a particularly enhanced risk of cardiovascular disease.

**Objective:**

In this secondary analysis of mActive-Smoke, a 12-week observational study, we investigated adherence to guideline-recommended moderate-to-vigorous physical activity (MVPA) in smokers and its association with the urge to smoke.

**Methods:**

We enrolled 60 active smokers (≥3 cigarettes per day) and recorded continuous step counts with the Fitbit Charge HR. MVPA was defined as a cadence of greater than or equal to 100 steps per minute. Participants were prompted to report instantaneous smoking urges via text message 3 times a day on a Likert scale from 1 to 9. We used a mixed effects linear model for repeated measures, controlling for demographics and baseline activity level, to investigate the association between MVPA and urge.

**Results:**

A total of 53 participants (mean age 40 [SD 12] years, 57% [30/53] women, 49% [26/53] nonwhite, and 38% [20/53] obese) recorded 6 to 12 weeks of data. Data from 3633 person-days were analyzed, with a mean of 69 days per participant. Among all participants, median daily MVPA was 6 min (IQR 2-13), which differed by sex (12 min [IQR 3-20] for men vs 3.5 min [IQR 1-9] for women; *P*=.004) and BMI (2.5 min [IQR 1-8.3] for obese vs 10 min [IQR 3-15] for nonobese; *P*=.04). The median total MVPA minutes per week was 80 (IQR 31-162). Only 10% (5/51; 95% CI 4% to 22%) of participants met national guidelines of 150 min per week of MVPA on at least 50% of weeks. Adjusted models showed no association between the number of MVPA minutes per day and mean daily smoking urge (*P*=.72).

**Conclusions:**

The prevalence of MVPA was low in adult smokers who rarely met national guidelines for MVPA. Given the poor physical activity attainment in smokers, more work is required to enhance physical activity in this population.

## Introduction

### Background

Smoking cessation and physical activity both lead to significant improvements in health [1]. Although smoking rates are decreasing because of regulation and taxation, behavioral counseling, and pharmacotherapy [1], an individual’s attempts to quit smoking are still challenging [2]. Furthermore, because a sedentary lifestyle increases cardiovascular risk [3], inactive smokers have a particularly enhanced risk of cardiovascular disease. The 2018 US Physical Activity Guidelines recommend greater than or equal to 150 min per week moderate activity or greater than or equal to 75 min per week vigorous physical activity (VPA), accumulated over bouts of any duration [4]. However, the prevalence of meeting these activity guidelines in the general adult population is unsatisfactory, with half of the US adults attaining fewer than 150 min of moderate-to-vigorous physical activity (MVPA) during leisure time per week, by self-report [4]. Moreover, 2 studies in young adults [5] and youth aged 14 to 18 years [6] found that self-reported attainment of physical activity guidelines was positively associated with noncigarette forms of tobacco use (eg, electronic cigarettes) but inversely associated with cigarette smoking, suggesting that physical inactivity and cigarette smoking may be compounding risk factors.

Physical activity has been suggested as an aid for smoking cessation, potentially through moderation of cravings and prevention of weight gain [7,8], but evidence is conflicting. Although there is insufficient evidence to recommend exercise as an aid for smoking cessation [7,9], previous meta-analyses suggested that acute bouts of exercise decrease urges, with activities at moderate to vigorous intensity having the greatest effect [10,11]. In addition, a study on active smokers found that a higher level of habitual MVPA was significantly associated with lower smoking urges [12]. However, this study used 7-day physical activity recall for assessing the levels of MVPA, leading to potential recall bias.

Methodological limitations such as recall bias and poor ecological validity are common in prior studies of exercise and smoking. Self-report for physical activity has been shown to poorly correlate with accelerometer data in accurately measuring MVPA and sedentary time, whereas ecological momentary assessment using mobile health (mHealth) tools, designed to sample real-time behaviors and experiences in the natural environment, performed better [13].

### Objectives

The goal of mActive-Smoke, a 12-week prospective observational study, was to assess the day-level association between objectively measured physical activity and concurrent smoking urges. Previously reported primary results [14] demonstrate that acute bouts of physical activity (ie, number of steps accumulated in a 30-min period before urge reporting), but not total daily steps, were associated with a modest decrease in smoking urges. Although we previously found a temporal association between acute bouts of activity and decreased urge, it is unclear whether the intensity of daily activity is associated with daily urge. In addition, despite well-established contributions of physical inactivity [3] and smoking on cardiovascular risk, there is limited research describing adherence to physical activity guidelines in adult smokers, with prior research mostly reliant on self-reported data. Thus, in this secondary analysis, we used prospective, objective measures to investigate adherence to guideline-recommended MVPA and the association between the intensity of physical activity and the urge to smoke.

## Methods

### Study Aim and Design

The aims of this secondary analysis were to report adherence to physical activity guidelines among smokers in the mActive-Smoke study population and to investigate the relationship between the intensity of physical activity and the urge to smoke. The methods for this 12-week prospective observational study have been previously reported [14], and a summary is provided below (Recruitment and Measurement of Baseline Variables and Data Collection). This study was approved by the Johns Hopkins School of Medicine Institutional Review Board.

### Recruitment and Measurement of Baseline Variables

We recruited 60 participants from April 7, 2016, to September 2, 2016, using on-site advertisements, social media, and physician referrals. Participants met inclusion criteria if they were aged 18 years or older, smoked at least 3 cigarettes per day on average, owned a smartphone, and were able to perform normal physical activity. Participants were screened for eligibility via email. At an initial meeting with a study coordinator, participants completed an enrollment questionnaire to record demographic characteristics, self-reported BMI (weight [kg]/height [m^2^]), physical activity, and smoking behavior. Baseline physical activity was assessed by the International Physical Activity Questionnaire (IPAQ)-short form, a questionnaire assessing walking time, sedentary time, and MVPA time in the past 7 days [15]. A *high* IPAQ score is defined as the equivalent of either VPA on 3 days or more per week at greater than or equal to 1500 metabolic equivalent of task (MET) minutes per week or 5 days or more per week of any combination of MVPA meeting greater than or equal to 3000 MET minutes per week [16]. Baseline smoking behavior was assessed with the Arizona Smoking Assessment Questionnaire [17].

### Data Collection

For the measurement of physical activity, participants used the Fitbit Charge HR (Fitbit Inc), a wrist-worn triaxial digital accelerometer allowing continuous monitoring of activity and heart rate. Patients were not instructed to alter their physical activity, but they could access step counts via the Fitbit mobile app (Fitbit Inc). Data from the Fitbit, including steps and the Fitbit-generated intensity level, were compiled in Fitabase, a secure research platform that collects real-time data from activity tracking devices [18]. Day-level and minute-level data were downloaded from Fitabase for each participant.

To measure smoking urges, an automated messaging service sent SMS text messages to participants 3 times per day, requesting that they respond with their instantaneous urge to smoke on a 9-point Likert scale from low to high. These messages were sent at participant-defined times, corresponding roughly to waking up, lunchtime, and returning home at the end of a day.

Participants were asked to complete a Web-based end-of-study survey regarding the study experience and their perceptions on physical activity and smoking urges. Survey questions and results were previously reported [14].

### Statistical Analysis

Baseline characteristics were summarized using descriptive statistics, frequency for categorical data and mean (SD) and median (IQR) for continuous data. Spearman and Pearson correlation coefficients were used for associations between variables.

As participants were not required to wear Fitbits during sleep, we defined nonwear time as 90 consecutive minutes of missing heart rate data between the hours of 10 am and 10 pm. Days with 2 or more 90-min nonwear periods and wear time of fewer than 6 hours within the target time window were excluded [19]. At least 6 total weeks of recorded data were required for inclusion. For the calculation of the prevalence of meeting weekly MVPA goals, we included weeks for which participants contributed 4 or more days of complete data [20].

Fitbit assigns minute-level activity into 4 intensity levels (0: *sedentary*, 1: *light*, 2: *moderate*, and 3: *vigorous*) [18]. We eschewed this measure of intensity given the proprietary algorithms and concern about accuracy [21,22] and used cadence as a surrogate measure of intensity. We elected to not include heart rate because of concerns about the accuracy of Fitbit’s heart rate measurement, particularly at vigorous intensities [23]. However, to explore the nature of Fitbit’s intensity variable, we compare daily MVPA minutes by Fitbit intensity levels (number of minutes spent at intensity level 2 or 3) with daily MVPA minutes by cadence threshold.

Cadence (steps per minute) is associated with objectively measured speed and intensity under controlled experimental conditions [24]. A threshold of 100 steps per minute is an evidence-based value generally associated with moderate intensity or greater than or equal to 3 METs and is best described as brisk walking, whereas a threshold of 130 steps per minute is associated with vigorous intensity or greater than or equal to 6 METs [25]. We created 4 cadence categories defined as 0 steps per minute (*no movement*), 1 to 59 steps per minute (*incidental movement to purposeful steps*), 60 to 99 steps per minute (*slow to medium walking*), and greater than or equal to 100 steps per minute (*brisk walking and faster*), whereas VPA was defined as cadence of greater than or equal to 130 steps per minute [25]. Daily minutes of MVPA were calculated by summing the minutes spent at cadence of greater than or equal to 100 steps per minute, and daily minutes of VPA was calculated by summing the minutes spent at cadence of greater than or equal to 130 steps per minute. We also calculated moderate+2×VPA, which weights moderate activity as 1 min and vigorous activity as 2 min, in accordance with physical activity guidelines [20], but as the results remained similar, we reported only MVPA and VPA.

Given the positively skewed data, we described MVPA minutes using median (IQR) and used the Wilcoxon rank sum test for comparing between-group differences. We reported the within-person and between-person prevalence of physical activity guideline adherence. We also estimated the prevalence of adherence with obtaining at least 150 min of MVPA or 75 min of VPA per week on greater than or equal to 50% of the study weeks. Although the physical activity guidelines were developed based on self-report, we opted to apply the physical activity guidelines to cadence measurements to provide a clinical context for the objective data. Daily urge to smoke was described using mean (SD) of the 3 to 4 urge messages sent each day. The mean daily urge was normally distributed and was treated as a continuous variable.

A repeated measures multivariable mixed effects linear model, accounting for autoregression and heteroscedasticity, was used to evaluate the relationship between daily MVPA minutes and daily urge. We adjusted for age, sex, race, education, BMI, baseline cigarettes per day, and baseline physical activity, which were selected a priori. Baseline physical activity was defined as a high level of activity or not by IPAQ. We explored interactions between age, sex, obesity status, baseline physical activity, and baseline cigarettes per day with daily MVPA minutes, with *P*<.10 considered evidence of interaction. The analysis was conducted using Stata (version 15·1; StataCorp).

## Results

### Study Flow and Baseline Characteristics

The study flow diagram, baseline characteristics, and survey results have been previously reported [14]. In brief, 60 participants were enrolled, and 53 participants recorded at least 6 weeks of data and were thus included in this analysis. In addition, 45 participants recorded 12 weeks of data, and 8 participants recorded 6 to 12 weeks of data. Of all participant-weeks, 80.1% (144/723) of weeks included at least 4 days of complete data. Participants sent a mean of 290 (SD 62) SMS text messages quantifying the urge to smoke. Moreover, 49 participants completed the Web-based exit survey. After excluding days using nonwear criteria, data from 3633 days were analyzed, with a mean of 69 days of data contributed by each participant. The mean age was 40 (SD 12) years, with 57% (30/53) women, 49% (26/53) nonwhite participants, and 30% (16/53) with a bachelor’s degree or higher. In addition, 40% (21/53) of participants were overweight, 38% (20/53) were obese, and 53% (28/53) had a high level of baseline activity as assessed by IPAQ (Table 1).

**Table 1:** Baseline characteristics of mActive-Smoke participants (N=53).

Characteristic		Participants, n (%)
**Sex**		
	Men	23 (43)
	Women	30 (57)
**Age (years)**		
	22-29	11 (21)
	30-39	16 (30)
	40-49	15 (28)
	50-59	7 (13)
	60-65	4 (8)
**Race**		
	White	27 (51)
	Nonwhite	26 (49)
**Education**		
	High school diploma or less	8 (15)
	Associate degree or some college	29 (55)
	Bachelor’s degree or higher	16 (30)
**BMI (kg/m^2^)**		
	<25.0 (normal or underweight)	12 (23)
	25.0-29.9 (overweight)	21 (40)
	≥30.0 (obese)	20 (38)
**International Physical Activity Questionnaire^a^**		
	Low	6 (11)
	Moderate	19 (36)
	High	28 (53)
**Cigarettes smoked per day**		
	≤10	34 (64)
	>10	19 (36)

^a^Categories defined by International Physical Activity Questionnaire guidelines.

### Patterns of Physical Activity

Participants accumulated a median of 7807 steps per day (IQR 5383-10,824). Of 53 participants, 31 (58%) met the recommended 30 min per day of MVPA on 1 or more days over the study duration. Of these 31 participants, the 30 min per day MVPA goal was met on a mean of 19% of days. Prevalence of adherence to national physical activity guidelines, defined as the proportion of participants who obtained at least 150 min per week of MVPA on at least 50% of total weeks, was 10% (5/51; 95% CI 4% to 21%). No participants attained at least 75 min per week of VPA on at least 50% of total weeks. Of the 53 participants, only 15 (28%) met 150 min per week of MVPA at least once throughout the study, and those 15 participants met that goal on a mean of 36% of weeks.

Overall, the median total MVPA minutes per week was 80 (IQR 31-162). Among all participants, median daily MVPA (more than 100 steps per min) was 6 min (IQR 2-13), whereas median daily minutes spent in lighter activity (60-99 steps per min) was 23 min (IQR 17-24). The median number of minutes spent in MVPA was significantly higher among men (12 min, IQR 3-20) than women (3.5 min, IQR 1-9; *P*=.004). The median number of minutes spent in lighter activity was also significantly higher among men (34 min, IQR 26-52) than women (18 min, IQR 15-23; *P*<.001). When comparing obese and nonobese participants, only MVPA minutes were significantly different, with median of 10 min (IQR 3-15) in participants with BMI less than 30 kg/m^2^ versus median of 2.5 min (IQR 1-8.3) in participants with BMI greater than or equal to 30 kg/m^2^ (*P*=.04; Table 2). There was poor correlation between the median daily MVPA minutes and baseline activity level (low, moderate, or high) as assessed by IPAQ (Spearman coefficient=−0.162; *P*=.25).

**Table 2:** Daily minutes spent at cadence bands by demographic characteristics over the study duration.

Characteristic		60 to 99 steps per minute, median (IQR)	More than 100 steps per minute, median (IQR)
**Sex**			
	Men	34 (26-52)^a^	12 (3-20)^a^
	Women	18 (15-23)^a^	3.5 (1-9)^a^
**Age (years)**			
	<40	28.5 (17-44.3)	6.25 (2-15.5)
	≥40	22.5 (18-29)	5 (2-12)
**BMI (kg/m^2^)**			
	<30	29 (18-44.5)	10 (3-15)^b^
	≥30	21 (15.5-28)	2.5 (1-8.3)^b^

^a^*P*<.01, two-sample Wilcoxon rank sum test.

^b^*P*<.05, two-sample Wilcoxon rank sum test.

### Association Between Day-Level Intensity and Urge

**Figure 1:**
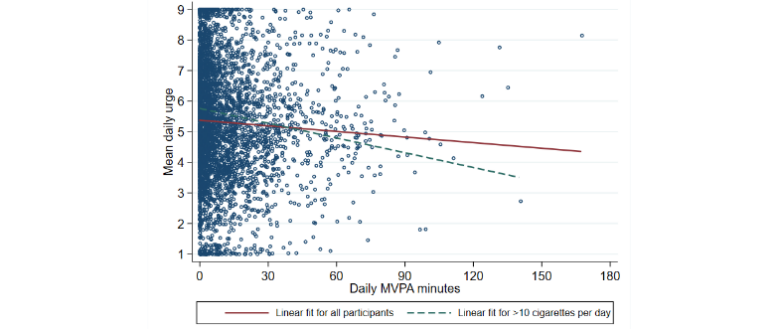
Mean daily smoking urge plotted against daily minutes of moderate-to-vigorous physical activity for all participants (N=53; 3633 person-days). MVPA: moderate-to-vigorous physical activity.

The number of daily MVPA minutes was positively skewed, with no clear association with urge upon inspection (Figure 1). Furthermore, logarithmic transformation of the MVPA variable did not reveal any clear relation with mean daily urge. There was no significant association between daily MVPA minutes and mean daily urge to smoke in either the unadjusted model (*P*=.74) or the adjusted model (*P*=.72).

We explored the interaction of daily MVPA minutes with binary demographic factors, defined as age greater than or equal to 40 years, sex, BMI greater than or equal to 30 kg/m^2^, and baseline high activity based on IPAQ. The *P* values for interaction are as follows: .41 for age, .15 for sex, .90 for BMI, and .32 for high activity. Thus, no interaction terms were included in the adjusted models.

### Sensitivity Analysis

Given that our prior work validating the urge to smoke revealed a positive association between mean urge over the course of the study and the number of cigarettes per day reported at the end of the study [14], we explored associations by baseline cigarette consumption. When stratifying by baseline cigarettes per day, those who smoked more than 10 cigarettes per day (n=19; 1333 person-days) had 0.293 lower daily urge per 30 min per day of MVPA (*P*=.03; 95% CI −0.563 to −0.023; Figure 1), but this was not significant on stratifying by greater than equal to 15 (n=16; 1100 person-days; *P*=.80) or greater than equal to 20 cigarettes per day (n=11; 809 person-days; *P*=.88).

### Comparison of Cadence Versus Fitbit’s Intensity Levels

**Figure 2:**
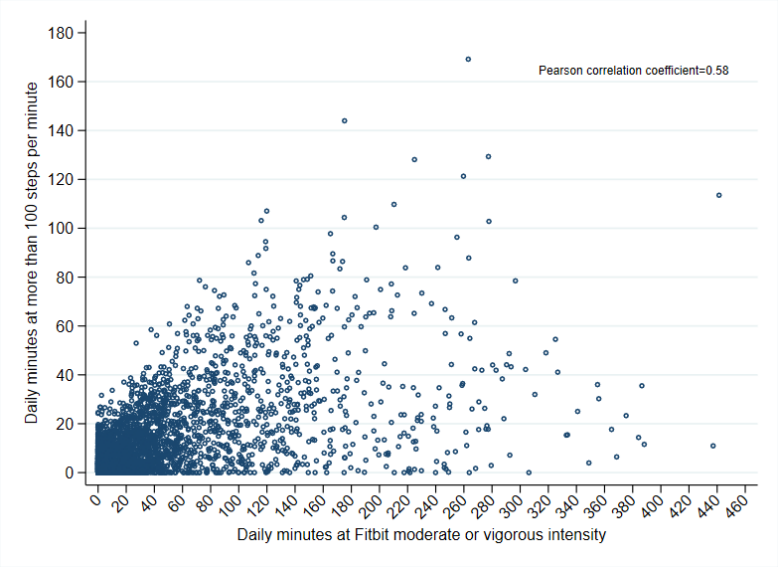
Daily moderate-to-vigorous physical activity (MVPA) minutes calculated using cadence thresholds versus daily MVPA minutes calculated using Fitbit’s intensity levels.

To elucidate the nature of Fitbit’s intensity variable, we compared the distribution of daily MVPA minutes calculated using the definition of cadence greater than or equal to 100 steps per minute with the distribution of daily MVPA minutes as defined by the number of minutes spent at *moderate-* or *vigorous*-intensity levels as defined by Fitbit’s algorithm (Figure 2). Although there was some correlation between daily MVPA minutes by cadence and daily MVPA minutes by Fitbit intensity (Pearson coefficient=0.58), the minutes categorized by Fitbit as MVPA tended to have lower cadences than the threshold of 100 steps per minute. The minutes that Fitbit categorized as *moderate* intensity had a median of 41 steps accumulated within that minute (IQR 14-67, range 0-138), whereas minutes categorized as *vigorous* intensity had a median of 95 steps accumulated (IQR 57-106, range 0-215). This led to a wider spread of daily MVPA minutes by Fitbit intensity (range 0-451 min) than by cadence (range 0-167 min).

## Discussion

### Principal Findings

In this secondary analysis of data from mActive-Smoke, we described intensity of physical activity in free-living adult smokers and found that the prevalence of MVPA was low, with 10% (5/51) of participants attaining greater than or equal to 150 min of MVPA on at least 50% of study weeks and no participants attaining greater than or equal to 75 min of VPA on at least 50% of study weeks. Overall, the median daily MVPA was 6 min, and this differed by sex and BMI. Most participants achieved at least 30 min per day of light-intensity activity (60-99 steps per minute) over the study duration. In regression analyses, there was no association between daily MVPA minutes and mean daily smoking urges among all participants. In addition, this study provides exploratory insights on using Fitbit’s intensity level to determine MVPA, compared with accepted cadence thresholds, which is a simpler marker of intensity available across measurement devices.

### Comparison With Prior Work

This study highlights the low prevalence of MVPA in adult smokers in a free-living environment and poor adherence to the 2018 US Physical Activity Guidelines of greater than or equal to 150 min per week of MVPA. Our results corroborate observations by prior analyses of accelerometer data from the National Health and Nutrition Examination Survey (NHANES), which are representative of the general US population. Using 2005 to 2006 NHANES data, Tucker et al [20] found that 59.6% of adults met the 2008 US Physical Activity Guidelines by self-report, whereas 8.6% of adults met the guidelines by accelerometer measurement, using the goal of greater than or equal to 150 min per week of MVPA in the 10-min bout. In the 2018 US Physical Activity Guidelines, the 10-min bout requirement was removed; thus, we did not include bouts in the calculation of MVPA minutes. Doing so would likely further reduce the estimated attainment of recommended physical activity levels and, as such, would not impact the conclusion of low levels of physical activity guideline adherence.

Another analysis of 2005 to 2006 NHANES data found that US adults accumulated only about 7 min per day of self-selected activity at a cadence of greater than or equal to 100 steps per minute (generally defined as MVPA), but the participants did accumulate, on average, about 30 min per day of activity at cadences of more than 60 steps per minute [26]. The NHANES dataset from these prior studies included accelerometer data over 1 to 7 days, whereas our data were obtained over 6 to 12 weeks [20,26], thus suggesting that low levels of physical activity may persist over time. These results highlight a need for further work in promoting physical activity in smokers to mitigate the compounding cardiovascular risk factors of inactivity and smoking.

Although there was no intervention to increase physical activity, 82% (40/49) of participants reported in the exit survey that they believed the study helped increase their physical activity [14], affirming the potential of mHealth tools and self-monitoring in promoting behavioral change. Furthermore, the 2018 Physical Activity Guidelines recommend information technology and mHealth interventions as a future direction for tracking and promoting physical activity [4].

This study also addresses the recall bias in prior studies on physical activity and smoking urges. For example, Haasova et al [12] showed that more habitual MVPA minutes based on 7-day activity recall significantly correlated with decreased urge over the past 7 days in 98 smokers. The difference between results from this 7-day recall study and our analysis suggests that the prevalence of MVPA and granularity of data are important factors to consider in studies on physical activity and smoking urge. Specifically, median daily MVPA based on 7-day recall was 45 min (IQR 17-77) in the study by Hassova et al [12], whereas we measured median daily MVPA over 12 weeks to be 6 min (IQR 2-13) using minute-level and day-level granularity of data. This is unsurprising given that this analysis and prior studies [13] found poor correlation between self-reported intensity via IPAQ and accelerometer-measured intensity.

This study analyzed intensity on a day level by quantifying the total daily minutes of MVPA for each person-day, which builds on prior studies showing that short bouts of MVPA acutely decrease cigarette cravings in a controlled laboratory setting [11]. In addition, we build on our primary analysis of mActive-Smoke, which showed that increased rate of step accumulation within 15-, 30-, or 60-min time windows before urge reporting was associated with decreased urge. When comparing our results with these prior studies on acute effects, we conclude that although MVPA may modestly decrease the urge to smoke immediately after physical activity, the effect of MVPA on the urge to smoke does not appear to persist throughout the day.

### Limitations

We acknowledge that this was a relatively small, single-center study, not generalizable to smokers everywhere. Despite the low prevalence of MVPA, the participants in this study had fairly high total daily step counts, suggesting that these participants may be active throughout the day at lighter intensities. This confers cardiovascular health benefits over a more sedentary lifestyle but may not be enough to affect smoking urges [4]. In addition, there is inherent selection bias, as participants who enrolled in this study were more likely to have an interest in behavior change, and step counts and urge reporting may be subject to the Hawthorne effect. However, it is important to note that even as part of a research study, the physical activity observed was low and similar to prior studies of the general population.

As this was a post hoc analysis, the sample size was not powered to test correlations between intensity and smoking urge. Although the total number of observations was high, the number of participants in this pilot study was relatively small, especially in the stratified models. However, we did account for repeated measures and autoregression in the model, and our smallest stratified group contained 809 person-days.

In addition, mActive-Smoke participants were lighter smokers, with 36% (19/53) reporting more than 10 cigarettes per day at baseline. Prior studies generally used a minimum of 10 cigarettes per day as the threshold for study inclusion [9,12]. We did not collect data on the time since the last cigarette to avoid overburdening participants with text messages; thus, we could not adjust for the potential confounding effect of recent smoking on urge. These factors may have generated a flooring effect, as physical activity may be less able to further reduce smoking urge when the urge is already low, either from a recent cigarette or because of lighter smokers having lower urges. We previously validated the urge scale and found that self-reported urges correlated well with daily cigarette consumption.

Cadence is an imprecise marker of intensity, correlating well with caloric expenditure, but does not account for types of activity other than walking or running, leading to possible underestimation of MVPA. In addition, the cadence thresholds used in these analyses were not adjusted for stride length variation among participants. Bias from lack of stride length adjustment is likely to be minimal, as overestimation of MVPA in participants with shorter stride is partially offset by underestimation of MVPA in participants with longer stride. Although the Fitbit Charge HR reports minute-level intensity levels, METs, and heart rate, we opted for cadence as the measure of intensity, as it is less dependent on other factors such as resting heart rate, comorbidities, and medications. Furthermore, validation studies have raised concerns about the accuracy of Fitbit’s reporting of heart rate [23], intensity, and energy expenditure, although step count was generally accurate [21,27].

Despite the advantages of objective activity measures, it is important to note that physical activity guidelines were developed based on self-reported data, and there are currently no guidelines based on accelerometer data. Linking objective measures with physical activity guideline attainment to provide clinical context has been done previously [20], but this method requires further validation in future studies. More work is needed to develop guidelines based on objective metrics of physical activity. Future directions include devising the optimal method of incorporating heart rate data into measurement of MVPA while accounting for medications and clinical characteristics. Finally, Fitbit provides other information about health behaviors, such as sleep, which could impact both physical activity and smoking urge and warrants further exploration.

### Conclusions

In this 12-week observational study of adult smokers using mHealth tools for real-time assessment of physical activity and smoking urge, the prevalence of MVPA was low, and participants rarely met national guidelines for physical activity. We found no day-averaged association between intensity of activity and smoking urges. On the basis of the known benefits of physical activity and the low levels observed in this study, more work is needed to address physical activity promotion in smokers.

## Abbreviations

IPAQ: International Physical Activity Questionnaire
MET: metabolic equivalent of task
mHealth: mobile health
MVPA: moderate-to-vigorous physical activity
NHANES: National Health and Nutrition Examination Survey
VPA: vigorous physical activity
